# Supplementary material for: Predicting Compressive Strength and Hydration Products of Calcium Aluminate Cement Using Data-Driven Approach
Source: Materials (Basel). 2023 Jan 9;16(2):654. doi: 10.3390/ma16020654 (PMC9861258; doi:10.3390/ma16020654)
Supplement: Supplementary file 1 [file materials-16-00654-s001.zip › materials-2145065-supplementary (1).pdf]

Predicting Compressive Strength and Hydration Products of Calcium Aluminate Cement Using Data-driven Approach

Sai Akshay Ponduru[1], Taihao Han[1], Jie Huang[2], and Aditya Kumar[1*]

1. Department of Materials Science and Engineering, Missouri University of Science and Technology, Rolla, MO, USA 65409
2. Department of Electrical and Computer Engineering, Missouri University of Science and Technology, Rolla, MO, USA 65409

***Corresponding Author**

Aditya Kumar

Associate Professor, Department of Materials Science and Engineering

Missouri University of Science and Technology

B49 McNutt Hall, 1400 N Bishop, Rolla, MO 65409

Email: kumarad@mst.edu; Phone: 573-341-6994; Fax: 573-341-6934

Table S1 presents the compressive strength, porosity, C_4AH_{19} content, straetlingite content, gibbsite content, and solid content in relation to mixture design and cement age.

Table S1: Mixture design detail of calcium aluminate cement corresponding to compressive strength, porosity, C_4AH_{19} content, straetlingite content, gibbsite content, and solid content.

Water-to-Cement Ratio (Unitless)	Cement Age (Hour)	Li_2CO_3 Content ($\%_{mass}$)	Measured Porosity ($\%_{vol}$)	Compressive Strength (MPa)	Calculated Porosity ($\%_{vol}$)	C_4AH_{19} Content ($\%_{vol}$)	Straetlingite Content ($\%_{vol}$)	Gibbsite Content ($\%_{vol}$)	Solid Content ($\%_{vol}$)
0.2	1	0	37.87	0	37.8624	0.798913	0.095486	0.172519	62.1376
0.2	2	0	37.93	0	37.9256	0.738643	0.088648	0.160558	62.0744
0.2	3	0	37.27	0.9	37.29242	1.342385	0.157145	0.280374	62.70758
0.2	4	0	35.84	1.3	35.82675	2.739904	0.3157	0.55772	64.17325
0.2	5	0	30.75	8.6	30.75708	7.573852	0.864131	1.517047	69.24292
0.2	6	0	20.1	14.1	20.13428	17.70273	2.013295	3.527186	79.86572
0.2	7	0	12.92	25.3	12.88896	24.61117	2.797086	4.898209	87.11104
0.2	8	0	10.47	32.5	10.45522	26.93175	3.060365	5.358742	89.54478
0.2	9	0	10.09	43.8	10.08353	27.28616	3.100574	5.429076	89.91647
0.2	24	0	7.49	57.5	7.459741	29.78795	3.384413	5.925573	92.54026
0.2	72	0	6.36	61.5	6.323343	30.87151	3.507347	6.140612	93.67666
0.2	168	0	3.84	64.5	3.874714	33.20629	3.772237	6.603963	96.12529
0.2	1	0.001	34.61	0	34.60606	3.903842	0.447753	0.788711	65.39394
0.2	2	0.001	33.45	0.3	33.44123	5.014513	0.573763	1.009131	66.55877
0.2	3	0.001	28.35	0.6	28.36418	9.855494	1.122993	1.969853	71.63582
0.2	4	0.001	24.23	2.5	24.23035	13.79712	1.570186	2.752093	75.76965
0.2	5	0.001	15.4	11	15.43394	22.18452	2.521772	4.416626	84.56606
0.2	6	0.001	14.94	28.8	14.92789	22.66704	2.576516	4.512385	85.07211
0.2	24	0.001	12.34	40	12.37551	25.10075	2.85263	4.995369	87.62449
0.2	72	0.001	9.94	47.9	9.934641	27.42813	3.116681	5.457251	90.06536
0.2	168	0.001	7.39	56.6	7.384206	29.85998	3.392584	5.939867	92.61579
0.2	1	0.003	37.2	0.3	37.22897	1.402888	0.16401	0.292381	62.77103
0.2	2	0.003	34.94	0.4	34.92817	3.596706	0.412907	0.727758	65.07183
0.2	3	0.003	34.56	1.9	34.54156	3.965341	0.45473	0.800916	65.45844
0.2	4	0.003	19.53	19.4	19.50078	18.30677	2.081826	3.647062	80.49922
0.2	5	0.003	14.17	35.3	14.20242	23.35879	2.654997	4.649665	85.79758
0.2	6	0.003	13.52	46.8	13.54692	23.98381	2.725909	4.773705	86.45308
0.2	24	0.003	10.47	59.9	10.52947	26.86096	3.052333	5.344693	89.47053
0.2	72	0.003	9.46	66.8	9.487212	27.85475	3.165083	5.541917	90.51279
0.2	168	0.003	6.69	73.6	6.702945	30.50956	3.466282	6.068781	93.29706
0.2	1	0.005	35.69	0.9	35.69869	2.862019	0.329554	0.581955	64.30131
0.2	2	0.005	34.15	2.8	34.15404	4.334841	0.496652	0.874245	65.84596
0.2	3	0.005	22.52	11.8	22.50667	15.44065	1.756652	3.078263	77.49333
0.2	4	0.005	15.52	22.9	15.50611	22.11571	2.513965	4.402968	84.49389

0.2	5	0.005	13.48	50.1	13.40092	24.12302	2.741703	4.801332	86.59908
0.2	6	0.005	10.33	68.7	10.30664	27.07342	3.076438	5.386858	89.69336
0.2	24	0.005	8.55	72.1	8.58896	28.71124	3.262255	5.711892	91.41104
0.2	72	0.005	6.59	74.5	6.627089	30.58189	3.474488	6.083135	93.37291
0.2	168	0.005	4.65	76.1	4.643495	32.47325	3.689071	6.458488	95.35651
0.2	1	0.007	32.5	1.3	32.52955	5.883798	0.672387	1.181646	67.47045
0.2	2	0.007	31.27	4.8	31.28423	7.071219	0.807105	1.417297	68.71577
0.2	3	0.007	21.61	14.8	21.60344	16.30188	1.854362	3.249179	78.39656
0.2	4	0.007	16.56	25.3	16.58515	21.08684	2.397236	4.198785	83.41485
0.2	5	0.007	12.73	46.4	12.74241	24.75091	2.812939	4.92594	87.25759
0.2	6	0.007	10.31	62.4	10.38095	27.00257	3.0684	5.372797	89.61905
0.2	24	0.007	8.12	72.1	8.13813	29.14111	3.311025	5.797203	91.86187
0.2	72	0.007	6.73	74.5	6.778768	30.43726	3.45808	6.054433	93.22123
0.2	168	0.007	5.61	76.1	5.638029	31.52496	3.581484	6.270293	94.36197
0.2	1	0.01	34.11	1.6	34.08937	4.396508	0.503648	0.886484	65.91063
0.2	2	0.01	24.38	12	24.36749	13.66635	1.55535	2.726141	75.63251
0.2	3	0.01	16.25	26	16.22621	21.42909	2.436065	4.266706	83.77379
0.2	4	0.01	13.35	33.9	13.32787	24.19267	2.749605	4.815155	86.67213
0.2	5	0.01	12.35	42.5	12.30204	25.17081	2.860579	5.009272	87.69796
0.2	6	0.01	10.6	45.8	10.60368	26.7902	3.044305	5.33065	89.39632
0.2	24	0.01	8.83	64.8	8.813949	28.49671	3.237916	5.669318	91.18605
0.2	72	0.01	6.79	72.2	6.85456	30.36499	3.449881	6.040091	93.14544
0.2	168	0.01	6.49	74.6	6.47528	30.72664	3.490911	6.111861	93.52472
0.25	1	0	41.86	0	41.88702	2.153362	0.249836	0.443283	58.11298
0.25	2	0	41.13	0	41.14195	2.875766	0.331797	0.586653	58.85805
0.25	3	0	40.9	0	40.912	3.098715	0.357092	0.6309	59.088
0.25	4	0	37.9	0.9	37.89272	6.026137	0.689227	1.211883	62.10728
0.25	5	0	33.72	5.4	33.73832	10.05414	1.146229	2.011292	66.26168
0.25	6	0	25.43	17	25.42071	18.11869	2.061204	3.611802	74.57929
0.25	7	0	15.53	36.9	15.54114	27.69768	3.148	5.512871	84.45886
0.25	8	0	13.4	44.4	13.38752	29.78578	3.384909	5.927281	86.61248
0.25	9	0	11.91	54.2	11.89143	31.23635	3.549484	6.215164	88.10857
0.25	24	0	8.86	58.9	8.860734	34.17484	3.882875	6.798344	91.13927
0.25	72	0	8.55	66.7	8.582634	34.44448	3.913467	6.851857	91.41737
0.25	168	0	9.16	68.9	9.1384	33.90562	3.85233	6.744914	90.8616
0.25	1	0.001	40.17	0	40.16242	3.825491	0.439549	0.775138	59.83758
0.25	2	0.001	39.7	0	39.69941	4.274412	0.490482	0.864232	60.30059
0.25	3	0.001	31.44	0.8	31.43856	12.28393	1.399213	2.45382	68.56144
0.25	4	0.001	22.37	12.1	22.36175	21.08458	2.397703	4.20042	77.63825
0.25	5	0.001	17.21	31	17.20539	26.08406	2.964925	5.192629	82.79461
0.25	6	0.001	15.11	48.9	15.13935	28.08725	3.192199	5.590186	84.86065

0.25	24	0.001	8.89	68.8	8.930191	34.10749	3.875234	6.784979	91.06981
0.25	72	0.001	6.9	71.1	6.904867	36.0712	4.098029	7.174701	93.09513
0.25	168	0.001	6.36	74	6.342095	36.61685	4.159936	7.282992	93.65791
0.25	1	0.003	39.11	0	39.11879	4.837366	0.554353	0.975957	60.88121
0.25	2	0.003	38.67	0	38.6528	5.289179	0.605614	1.065625	61.3472
0.25	3	0.003	32.06	0.9	32.04685	11.69415	1.332298	2.336771	67.95315
0.25	4	0.003	21.65	22.1	21.65296	21.77181	2.475673	4.336809	78.34704
0.25	5	0.003	20.54	25.4	20.55177	22.8395	2.596809	4.548705	79.44823
0.25	6	0.003	17.77	36.8	17.80064	25.50692	2.899445	5.078088	82.19936
0.25	24	0.003	9.84	60.1	9.830676	33.23441	3.776177	6.611704	90.16932
0.25	72	0.003	8.48	73.6	8.513041	34.51195	3.921123	6.865249	91.48696
0.25	168	0.003	7.82	77.3	7.815613	35.18816	3.997843	6.999451	92.18439
0.25	1	0.005	43.1	0.3	43.08338	0.993399	0.118231	0.213074	56.91662
0.25	2	0.005	35.39	2.7	35.41268	8.430722	0.962042	1.689103	64.58732
0.25	3	0.005	21.77	28.9	21.78205	21.64665	2.461473	4.311969	78.21795
0.25	4	0.005	16.05	32.1	16.07543	27.17964	3.089226	5.410061	83.92457
0.25	5	0.005	15.67	38.8	15.67487	27.56802	3.13329	5.487139	84.32513
0.25	6	0.005	15.19	40.4	15.20638	28.02226	3.184825	5.577288	84.79362
0.25	24	0.005	14.42	43.3	14.40032	28.80379	3.273495	5.732392	85.59968
0.25	72	0.005	13.01	47.4	12.98075	30.18017	3.429654	6.005552	87.01925
0.25	168	0.005	12.57	48	12.57305	30.57547	3.474504	6.084005	87.42695
0.25	1	0.007	40.84	1.2	40.79691	3.210309	0.369753	0.653047	59.20309
0.25	2	0.007	30.28	9.7	30.27667	13.41047	1.527026	2.677397	69.72333
0.25	3	0.007	18.64	22.7	18.65687	24.67675	2.805256	4.91333	81.34313
0.25	4	0.007	17.86	30.9	17.86666	25.44291	2.892183	5.065385	82.13334
0.25	5	0.007	17.57	38	17.60245	25.69908	2.921247	5.116225	82.39755
0.25	6	0.007	16.59	41.2	16.60809	26.66319	3.030631	5.307564	83.39191
0.25	24	0.007	15.02	44	15.00521	28.2173	3.206955	5.615997	84.99479
0.25	72	0.007	14.82	48.1	14.80382	28.41257	3.229109	5.654751	85.19618
0.25	168	0.007	9.42	67.7	9.415634	33.63682	3.821833	6.691568	90.58437
0.25	1	0.01	39.88	4.3	39.87319	4.105917	0.471365	0.830792	60.12681
0.25	2	0.01	31.94	9.4	31.92537	11.81193	1.345661	2.360146	68.07463
0.25	3	0.01	20.37	31.2	20.35671	23.02863	2.618267	4.58624	79.64329
0.25	4	0.01	14.48	40.3	14.46764	28.73852	3.26609	5.719439	85.53236
0.25	5	0.01	13.29	45.8	13.31979	29.85145	3.392359	5.940314	86.68021
0.25	6	0.01	12.64	50	12.64106	30.50952	3.467022	6.070917	87.35894
0.25	24	0.01	9.79	58.7	9.76157	33.30141	3.783779	6.625001	90.23843
0.25	72	0.01	9.36	71	9.346366	33.70398	3.829453	6.704897	90.65363
0.25	168	0.01	8.04	76.9	8.025128	34.98502	3.974795	6.959135	91.97487
0.3	1	0	47.34	0	47.35994	1.257822	0.148801	0.267194	52.64006
0.3	2	0	46.72	0	46.74064	1.866885	0.217904	0.388073	53.25936

0.3	3	0	44.77	0	44.76348	3.811354	0.43852	0.773988	55.23652
0.3	4	0	44.4	0	44.39658	4.172184	0.479459	0.845601	55.60342
0.3	5	0	41.95	0.9	41.96447	6.564084	0.750839	1.320316	58.03553
0.3	6	0	32.9	7.5	32.87214	15.50606	1.76538	3.09501	67.12786
0.3	7	0	27.79	20	27.7833	20.51076	2.333204	4.088282	72.2167
0.3	8	0	20.04	35.3	20.06343	28.10298	3.194603	5.595092	79.93657
0.3	9	0	17.91	48.7	17.92422	30.20681	3.4333	6.012635	82.07578
0.3	24	0	11.82	66.9	11.79077	36.23885	4.117684	7.209799	88.20923
0.3	72	0	10.16	75.1	10.19125	37.81193	4.296162	7.522004	89.80875
0.3	168	0	10.04	75.8	10.06266	37.93839	4.31051	7.547102	89.93734
0.3	1	0.001	44.05	0	44.08143	4.482126	0.514624	0.907114	55.91857
0.3	2	0.001	42.03	0	42.01773	6.5117	0.744896	1.309919	57.98227
0.3	3	0.001	38.81	0.5	38.79045	9.685612	1.105002	1.939838	61.20955
0.3	4	0.001	26.08	2.3	26.09127	22.17481	2.522003	4.418541	73.90873
0.3	5	0.001	25.77	7.8	25.79783	22.4634	2.554747	4.475817	74.20217
0.3	6	0.001	21.66	11.3	21.63556	26.55685	3.019182	5.288235	78.36444
0.3	24	0.001	15.13	47.3	15.13451	32.9504	3.744582	6.557148	84.86549
0.3	72	0.001	12.26	57.5	12.23606	35.80093	4.067998	7.122886	87.76394
0.3	168	0.001	5.72	68.6	5.700422	42.22849	4.797257	8.39855	94.29958
0.3	1	0.003	44.29	0	44.2916	4.27543	0.491173	0.866092	55.7084
0.3	2	0.003	37.99	0.7	37.97353	10.48903	1.196156	2.099291	62.02647
0.3	3	0.003	32.52	4.9	32.53391	15.8387	1.803121	3.161029	67.46609
0.3	4	0.003	25.03	11	25.03248	23.21609	2.640145	4.625202	74.96752
0.3	5	0.003	21.3	30.3	21.27403	26.9124	3.059521	5.358799	78.72597
0.3	6	0.003	19.6	36.7	19.5768	28.58157	3.248902	5.690076	80.4232
0.3	24	0.003	18.23	42.2	18.23145	29.90467	3.399019	5.952669	81.76855
0.3	72	0.003	11.39	62.5	11.4082	36.61509	4.160371	7.284471	88.5918
0.3	168	0.003	11.12	68.4	11.08876	36.92925	4.196015	7.346822	88.91124
0.3	1	0.005	42.34	0.2	42.33695	6.19776	0.709277	1.247612	57.66305
0.3	2	0.005	37.68	3.1	37.64561	10.81152	1.232746	2.163295	62.35439
0.3	3	0.005	27.62	21.2	27.60904	20.68213	2.352647	4.122293	72.39096
0.3	4	0.005	22.37	32.2	22.35633	25.848	2.938756	5.14755	77.64367
0.3	5	0.005	21.16	35	21.15335	27.03108	3.072987	5.382354	78.84665
0.3	6	0.005	18.16	40	18.17005	29.96506	3.40587	5.964654	81.82995
0.3	24	0.005	16.14	46.7	16.13153	31.96986	3.633332	6.362543	83.86847
0.3	72	0.005	16	48.8	16.00722	32.09212	3.647204	6.386808	83.99278
0.3	168	0.005	8.29	65.9	8.252586	39.71853	4.512482	7.900403	91.74741
0.3	1	0.007	42.19	0.5	42.17742	6.354653	0.727078	1.27875	57.82258
0.3	2	0.007	38.12	4.2	38.13724	10.32802	1.177889	2.067336	61.86276
0.3	3	0.007	24.71	19	24.67808	23.56463	2.679691	4.694376	75.32192
0.3	4	0.007	23.56	22.2	23.55092	24.67316	2.805462	4.914383	76.44908

0.3	5	0.007	22.32	33.9	22.29638	25.90695	2.945446	5.159251	77.70362
0.3	6	0.007	22.2	36.8	22.23641	25.96593	2.952137	5.170956	77.76359
0.3	24	0.007	16.36	54.1	16.31784	31.78663	3.612543	6.326178	83.68216
0.3	72	0.007	12.99	67.9	12.9968	35.05277	3.983113	6.9744	87.0032
0.3	168	0.007	9.14	71.2	9.095267	38.88978	4.418453	7.735924	90.90473
0.3	1	0.01	41.98	1.2	41.91118	6.616485	0.756785	1.330716	58.08882
0.3	2	0.01	32.87	7.8	32.92845	15.45069	1.759097	3.08402	67.07155
0.3	3	0.01	21.93	31.7	21.93625	26.26113	2.98563	5.229544	78.06375
0.3	4	0.01	17.75	38.7	17.73963	30.38836	3.453897	6.048665	82.26037
0.3	5	0.01	15.88	51.9	15.88281	32.21447	3.661085	6.41109	84.11719
0.3	6	0.01	14.64	58.6	14.63386	33.44277	3.800446	6.654868	85.36614
0.3	24	0.01	12.97	69.7	12.93353	35.11499	3.990173	6.986749	87.06647
0.3	72	0.01	11.63	70.1	11.59959	36.42687	4.139016	7.247115	88.40041
0.3	168	0.01	8.18	79.8	8.187599	39.78244	4.519733	7.913088	91.8124
